# The Outcome of Fixing Distal Femur Periprosthetic Fracture around Total Knee Replacement using a Locking Plate Non-Contact Bridging (NCB)

**DOI:** 10.5704/MOJ.2203.007

**Published:** 2022-03

**Authors:** MA Rahuma, H Noureddine

**Affiliations:** 1 Department of Trauma and Orthopaedics, Aintree University Hospitals NHS Foundation Trust, Liverpool, United Kingdom; 2 Department of Trauma and Orthopaedics, University Hospital Southampton NHS Foundation Trust, Southampton, United Kingdom

**Keywords:** distal femur periprosthetic fracture, total knee replacement, locking plate, non-contact bridging plate

## Abstract

**Introduction:**

Surgical fixation of peri-prosthetic distal femur fractures around knee replacements poses a challenge, especially in frail patients, with variable outcomes reported in the literature. This study looks at the outcomes of a consecutive series of patients presenting with such fractures and treated by using a locking plate fixation.

**Materials and methods:**

A total of 21 consecutive patients who were admitted to our trauma unit over 31 months and underwent fixation with the Non-Contact Polyaxial Locking plate system were retrospectively identified and their acute treatment with follow-up outcomes were analysed.

**Results:**

The mean age was 81 years and 71% were in ASA grades 3 and 4. Fracture morphologies were classified as per the Su classification, yielding 8 (38%) classified as Su one, 4 (19%) as Su two, and 9 (43%) as Su type three. Postoperatively, 2 patients (9.5%) died due to hospital-acquired pneumonia, and another 2 patients (9.5%) developed wound infections necessitating further return to theatre. Additionally, 2 (9.5%) patients had distal femoral replacements due to non-union. Mean discharge time was 28 days with 12 patients (55% of patients) starting protected weight-bearing six weeks after surgery.

**Conclusion:**

The incidence of morbidity, mortality was significant and re-operation was required in patients treated as described, and these were partly attributed to the patient’s average age and the pre-existing comorbidities. Significant variations were noted in the time to discharge, rehabilitation, and time to achieve fracture union. However, the majority of fractures did eventually unite. Patients with comminuted fractures and insufficient bone stock are more likely to progress to non-union and end up requiring revision knee arthroplasty.

## Introduction

The National Joint Registry (NJR) shows that 94,516 primary total knee replacements (TKR) were performed in the UK in 2019^1^, in an upward trend year on year since the NJR was established, reflecting the ageing population. Subsequently, the number of peri-prosthetic fractures around knee replacements is also rising and estimated to be around 0.3-2.5%^2^. Such fractures are challenging for both surgeon and patient, with treatment aiming to achieve early full weight-bearing and a stable painless joint without gross malalignment.

Historically a lot of these fractures were treated nonoperatively^3^ and this could be suitable for patients with undisplaced fractures or those with poor soft tissue envelopes or significant comorbidities, making the risk of surgery prohibitive. However, early surgical fixation of this type of fracture facilitates patient care and allows earlier mobilisation. The treatment method depends on the implant stability, location of the fracture and bone stock quality. Over the last decade, introducing locking plates offered a feasible option to achieve a stable construct in such fractures. Surgical management of peri-prosthetic distal femur fractures around total knee arthroplasties is often complicated. The preferable option is implant retention and surgical stabilisation of the fracture using a locking plate construct or retrograde nailing. An alternative is revision arthroplasty surgery, usually reserved for patients with loose implants, failure of primary fixation, or poor bone stock. Such cases are even more challenging in the presence of very distal fractures around the femoral component with a wellfixed implant.

Multiple classification systems have been suggested to describe those fractures, incorporating variables such as fracture morphology, prosthetic component stability and bone stock, among other factors. The authors elected to adopt the Su classification system as in contrast to other systems, it emphasises the distance between the fracture and the femoral component, which plays an integral role in dictating whether the surgical fixation is going to entail a locking plate, a retrograde nail, or a revision arthroplasty^4^. The authors designed this study to evaluate a consecutive series of patients presenting with peri-prosthetic supracondylar femoral fractures around a total knee replacement treated with the Non-Contact Bridging Polyaxial Locking Plate System (NCB Plate) fixation, specifically looking at complication rates, mortality, and length of hospital stay, time to achieve union, and fixation failures.

## Materials and Methods

All patients admitted to our trauma unit with distal femur fractures around a TKR over a period of 31 months from January 2016 to Jun 2018 were identified, yielding a total of 27 patients. Six patients were excluded, (open fracture one, pre-existing distal femur fixation 2, loose femoral implant 2 and a fracture occurring between a proximal femoral stem and TKR component one), resulting in a cohort of 21 patients with isolated first-time closed fractures around a TKR. Twenty-one underwent operative fixation using a noncontact bridging locking plate [NCB Zimmer TM]. Five different experienced consultant orthopaedic surgeons undertook the surgeries. The stability of the femoral implant was assessed pre-operatively using radiographic findings and confirmed clinically during surgery.

**Fig. 1:**
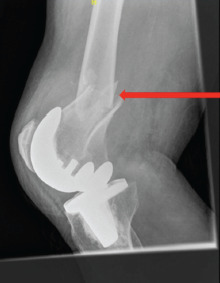
Su type two fracture with well-fixed implant. The fracture is proximal to the femoral component with adequate bone stock distally (arrow).

**Fig. 2:**
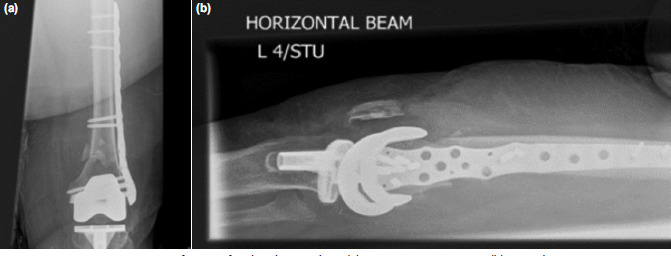
Post-operative Su type II fracture fixed with NCB plate. (a) Antero-posterior view. (b) Lateral view.

The fractures were reduced, and the adequacy of reduction was confirmed under direct vision and an image intensifier. The reduction was held with the plate system as shown in (Fig. 1 and 2). A mixture of bi-cortical and uni-cortical, locking screws, nonlocking screws and locked Dal-miles cables were used to maintain the fixation. No patient required structural allografts or cement augmentation. All patients received intravenous prophylactic antibiotics before skin incision and further three doses post-operatively over 24 hours. The plate's average working length was 5cm, and the average number of screws in the distal fragment was four.

## Results

In our series of twenty-one patients, the Mean age was 81 years (51-94) (Table I). The male to female ratio was (3:18), 71% of patients were ASA grade three or four. The average time from injury to the operation was 4.2 days (2-10 days). Reasons for delay included availability of appropriate surgeon, patients not fit for surgery, and theatre availability. The mean time from primary knee arthroplasty to injury was 7.6 years (3 – 17 years).

**Table I:** Characteristics and outcome of all cases

Age and Gender	ASA	Complication	Mal-union	Su Type	Mortality at 30 days	N0 of DLS
74y F	3			III	Yes	5
83y F	2	*		III		4
94y F	3			II	Yes	4
75y F	3	*		III		6
81y F	2		11^o^AA	I		6
93y F	3	X		I		5
82y F	3			I		5
94y F	3			III		4
92y F	2			III		5
79y F	3			II		4
70y F	3			I		4
79y M	3			II		4
85y F	4			I		4
92y F	3			I		4
88y F	4	X		I		4
75y F	3		11^o^AA	III		5
93y F	4			I		4
91y M	3			III		5
69y F	2			II		6
51y F	2			III		4
59y M	2			III		4

* = Non-union, X = Deep wound infection, AA = Anterior angulation, and DLS = Distal locking screws

Data on patient demographics, fracture pattern, ASA grade, surgical procedure, time to union, time to full weight-bearing status, complications, and 30-day mortality were collected retrospectively by reviewing the medical records. The implant is defined as radiologically loose when there is a radiolucent line between the implant and cement mantle which was not present on previous images. The fracture pattern was classified using the system described by Su^5^.

Physiotherapy was started in the immediate post-operative period, including a passive and active non-weight bearing range of movement exercises. The non-weight bearing mobilisation period extended for six weeks, then patients gradually progressed to full weight-bearing, depending on clinical and radiological evidence of bone healing. Patients were followed up at two weeks for wound checks, six weeks for radiographs and weight-bearing decision, then at three and six months for further radiographs. Patients had separate follow-up appointments with the physiotherapist when those were not feasible concomitantly with the clinic appointments.

The fracture was considered radiologically united when callus was visible across at least three cortices on both anteroposterior and lateral radiographs. Delayed union was defined as a fracture union that was not achieved after six months without an additional surgical procedure. A diagnosis of non-union was accepted in fractures with no progressive radiological bony healing features on three consecutive radiographs during the initial six months follow-up period and necessitating additional or revision procedures.

Three independent assessors were allocated to identify the time to radiological union. Mal-alignment or mal-reduction of the fracture was recorded in the coronal and sagittal planes, and mal-union was defined as more than 10° angulation in any plane.

Two patients died within the 30-day post-operative period (9.5%), secondary to hospital-acquired pneumonia (Table I). A further five patients died within 18 months post-surgery (23.8%). The operating surgeon suggested weight-bearing status based on radiological and clinical findings. Of these, 55% started protected weight-bearing (no restriction on the amount of weight put down but only for short distances and with the support of crutches/frame) six weeks after surgery. A physiotherapist saw each patient within 24 hours of the operation to start the early range of movement exercises. Patients were discharged from the hospital when deemed safe with adequate community social support where needed. Further surgery was required for four patients (19%). Of those, two patients (9.5%) underwent revision arthroplasty using long stem cemented prosthesis [Stanmore Modular Distal Femur Implant System] for non-union. The other two patients had a deep wound infection that required debridement, irrigation, and further antibiotic treatment. Both patients’ infections settled with no further sequelae. Both non-union cases were of the subgroup with fractures classified as Su three comprising (9/21),(42.8%), meaning that 22,2% of this subgroup (2/9) developed non-union.

The minimum number of screws in the distal fragment for Su two and three fractures was four. The mean time for discharge from the hospital was 28 days (7-39). Given the age and comorbidity population demographics, it was not easy to obtain the common function and knee pain scores. The final outcome was measured pragmatically as functionally pain-free, fully weight bearing on the operated knee as reported by the patient. This was achieved at a mean of 4.7 months (2.4-11 months) following surgery, 12 patients (58.2%) came from their own homes and were discharged back to their homes, 7 patients (33.3%) came from a nursing/residential home and were admitted back to the same institution and 2 patients (9.5%) were admitted from home and subsequently discharged to a nursing home.

## Discussion

Supracondylar femoral peri-prosthetic fractures are common injuries around a TKR, with an incidence of 0.3% to 2.5 % after primary TKR and 1.6% to 38% after revision TKR^6-10^. Tibial peri-prosthetic fractures are less common, with an incidence of 0.4% in the primary setting and a higher incidence in revision TKR^11^. A fracture of the distal femur above a TKR constitutes a difficult treatment dilemma. Some of the issues or challenges associated with it include a short distal segment for fixation, osteoporotic bone leading to the fractures' comminution, and varus collapse potential without both column support^2^.

Surgical options include intramedullary devices, external fixators, fixed-angle devices (blade plates, dynamic condylar screws), condylar buttress plates, and more recently, locking plates which can be placed in a sub-muscular manner. The distal segment's bone stock is considered a key limiting factor in obtaining adequate fixation. As such, Su *et al*^5^ proposed their classification system to guide treatment and decision-making. According to their classification, type one fractures are amenable to either retrograde or antegrade intramedullary nailing, and type two fractures require either retrograde intramedullary nailing or fixed-angle plating. On the other hand, type three fractures can be managed with a fixed-angle device or revision distal femoral arthroplasty when bone stock is poor or a loose femoral implant.

The systemic review by Ebraheim *et al* (2015)^7^ accumulated 345 patients with Lewis-Rorabeck type two fractures (displaced fracture with a stable prosthesis)^2^, the majority of whom were treated with either a locking plate (n=180) or intramedullary nailing (n=122) and recorded comparable successful healing rates of 87% and 84%, respectively^7^. However, the same review found that those with the same fracture subtype (calculated to be the most common) had a significant discrepancy in terms of the overall complication rate with those treated with a locking plate recording a 35% overall complication rate versus a 53% complication rate in their IM nailing counterparts, with complications including infection, metalwork failure, non-union, delay union, malunion and need for revision surgery^7^.

However, the systemic review by Herrera *et al* (2009), which spanned 415 distal femur periprosthetic fractures distributed across 29 published case series covering the period from 1981 till 2006, had different outcomes. Fifty-eight patients had intramedullary nailing, and 40 had locking plates, reflecting the historic nature of some of the treatments with a high percentage of non-operative treatment and nonlocking plating. Looking at the nailing and locking plate subgroups in isolation yields superior outcomes in the former. There was a 1.5% non-union rate in the nailing patients compared to 5.3% in the locking plating group. Similarly, the nailing group were superior when it came to infection rates and the need for revision surgery (0 vs 5.3% and 4.6 vs 8.8%, respectively)^8^.

This inconsistency in findings when attempting to decipher which method is superior is perhaps best reflected in the finding of the meta-analysis conducted by Shin *et al* (2017), which included eight studies, and found no statistical difference between both groups in terms of time to achieving union, non-union rates, and need for revision surgery^12^.

It is worth noting nonetheless that some of these results include plate options that predated the non-contact bridging locking plates. The NCB plate availability widened the range of indications for plate fixation in femoral fractures. The plate provides surgeons with the flexibility to optimise fixation by locking the screws at variable angles when working around comminuted distal femoral fragments. In our series, NCB plates were used in such cases with an average of five screws to purchase the distal fragments. Based on the outcomes we recommend a minimum of four distal locking screws. Finally, this is a retrospective case series with a relatively limited cohort, reflecting the scarcity of such cases. Furthermore, whilst all our surgeons adopt the same principles in treating such fractures, the fact remains that this is not a single operator series hence individual operator bias could not be fully excluded. Also, our study did not include a control group of patients treated with alternative methods, and we relied on comparing our outcomes with published data.

## Conclusion

There is a significant incidence of morbidity, mortality, and re-operation associated with periprosthetic distal femur fractures in this group of patients in part related to the preexisting past medical history, where more than 70% of patients in our study scored as ASA grade three and four. The time to discharge from the hospital and to reach full weightbearing status and union can be prolonged. However, the vast majority of fractures do eventually unite. We recommend that revision arthroplasty procedures be considered for the more comminuted Su type three fractures with insufficient bone stock rendering adequate fixation difficult.
